# Highly Efficient and Stable Mn-Co_1.29_Ni_1.71_O_4_ Electrocatalysts for Alkaline Water Electrolysis: Atomic Doping Strategy for Enhanced OER and HER Performance

**DOI:** 10.3390/molecules30051162

**Published:** 2025-03-05

**Authors:** Yijia Cheng, Xingyan Guo, Zhizheng Ma, Kehan Dong, Lihua Miao, Shuai Du

**Affiliations:** 1 School of Medical Information Engineering, Shenyang Medical College, Shenyang 110034, China; chengyijia0515@163.com (Y.C.); 17702475778@163.com (X.G.); zz865304207@163.com (Z.M.); 15041662742@163.com (K.D.); 2 School of Electronic Information Science and Technology, Liaoning University, Shenyang 110036, China; shuaidu@163.com

**Keywords:** overall water splitting, Mn-Co_1.29_Ni_1.71_O_4_, hydrogen evolution reaction, oxygen evolution reaction, overpotential

## Abstract

Water electrolysis for hydrogen production has garnered significant attention due to its advantages of high efficiency, environmental friendliness, and abundant resources. Developing cost-effective, efficient, and stable materials for water electrolysis is therefore crucial. In this work, we synthesized a series of highly efficient multifunctional Mn-Co_1.29_Ni_1.71_O_4_ electrocatalysts through an atomic doping strategy for alkaline electrocatalysts. The unique structure features and large specific surface area of these catalysts provide abundant active sites. The Mn-Co_1.29_Ni_1.71_O_4_ catalysts exhibit an excellent oxygen evolution reaction (OER) performance in 1.0 M KOH electrolyte, with an overpotential of 334.3 mV at a current density of 10 mA cm^−2^ and 373.3 mV at 30 mA cm^−2^. Additionally, the catalysts also demonstrate a Tafel slope of 76.7 mV dec^−1^ and outstanding durability. As hydrogen evolution reaction (HER) electrocatalysts, it shows an overpotential of 203.5 mV at −10 mA cm^−2^ and a Tafel slope of 113.6 mV dec^−1^. When the catalysts can be utilized for the overall water splitting, the catalyst requires a decomposition voltage of 1.96 V at 50 mA cm^−2^. These results indicate that the high catalytic activity and stability of Mn-Co_1.29_Ni_1.71_O_4_ samples make it a highly promising candidate for industrial-scale applications.

## 1. Introduction

In recent years, with the growing severity of energy and environmental issues, renewable and clean energy sources have garnered widespread attention [1,2,3]. Among these, hydrogen energy stands out due to its high energy density, pollution-free nature, wide range of applications, and easy accessibility, making it a highly promising new energy source [4]. Electrochemical water splitting is a novel technology with high energy conversion efficiency and mass activity, offering high efficiency and long-term durability at low overpotentials [5]. It leads to the particularly promising in the field of hydrogen energy. The oxygen evolution reaction (OER) and hydrogen evolution reaction (HER) are the two essential processes in electrochemical water splitting [5,6]. However, the high overpotential and slow kinetics of both reactions, especially the sluggish four-electron oxygen oxidation process, significantly hinder the practical application of water splitting. At the same time, the growing scarcity of freshwater resources highlights the abundance of seawater, which offers a promising alternative. However, chloride ions (Cl^−^) present in seawater can lead to chlorine oxidation reactions at the anode and strongly corrode the electrode and substrate materials [7]. Therefore, achieving optimal OER and HER performance in alkaline seawater electrolysis environments remains challenging. Nevertheless, certain materials not only maintain their OER performance but also exhibit significantly enhanced HER activity. This underscores the urgent need for the development of bifunctional electrocatalysts with high catalytic activity and stability to improve water-splitting efficiency for both HER and OER.

Currently, noble metals such as Pt/C, RuO_2_, and IrO_2_ are widely recognized as excellent catalysts for HER and OER. However, their limited availability and high cost severely restrict their large-scale application [8,9,10]. Therefore, it is crucial to develop low-cost, abundant electrode materials with high catalytic activity and stability. Transition metal nickel–cobalt oxides (Ni–Co oxides) have attracted extensive attention for their alkaline OER electrocatalytic activity [11]. NiO can be ascribed as a primary focus due to its face-centered cubic atomic arrangement and favorable surface characteristics, with Ni^3+^ and Ni^2+^ sites promoting the adsorption of O^2^⁻ and O^−^ ions. Ksenia Fominykh and colleagues synthesized NiO nanoparticles smaller than 5 nm via a hydrothermal method. The surface oxidation to Ni^3+^ resulted in excellent OER activity, with an overpotential of 320 mV required to reach a current density of 10 mA cm^−2^ [12]. Yuan et al. [13] successfully synthesized 3D interconnected NiO nanosheets supported by pristine graphene using an in situ self-assembly method, achieving an overpotential of 320 mV at 10 mA cm^−2^ and a Tafel slope of 52.4 mV dec^−1^, the lowest reported for NiO-based catalysts. However, transition metal oxides typically suffer from poor conductivity, limiting their electrocatalytic activity. Elemental doping has been shown to enhance both OER and HER activities by optimizing the local crystal and electronic structures.

Manganese (Mn) doping has recently garnered significant attention in the study of nickel–cobalt oxides, as it can substantially improve their structure and electrocatalytic activity [14]. Nickel–cobalt oxides are valued for their excellent mechanical and thermal stability, as well as good electrical conductivity [15]. The introduction of Mn further enhances the number of active catalytic sites and improves conductivity while optimizing the adsorption energy of reaction intermediates. For example, Mn doping in Ni–Co oxides exposes more active sites, improves electron conductivity, and enhances the electrocatalyst’s performance in both HER and OER by adjusting hydrogen and water adsorption energies [16]. Studies show that Mn doping also fine-tunes the electronic and crystal structures of Ni–Co oxides, optimizing the adsorption of oxygen-containing intermediates and accelerating oxygen generation. Specifically, Mn doping in Ni–Co oxides effectively modulates the water adsorption-free energy at different catalytic sites and generates more low-coordination, defect-rich active sites, thus improving catalytic efficiency. As such, Mn-doped Ni–Co oxides present a promising modification strategy for electrocatalysts, showing significant potential in oxygen evolution reactions [17,18].

In this work, we successfully synthesized Mn-Co_1.29_Ni_1.71_O_4_ nanomaterials on nickel foam substrates via a hydrothermal method. By controlling the Mn doping content, we were able to tune the electrochemical properties of Mn-Co_1.29_Ni_1.71_O_4_. The catalyst exhibited high OER efficiency in 1.0 M KOH electrolyte, with an overpotential of 373.3 mV at 30 mA cm^−2^ and a Tafel slope of 76.7 mV dec^−1^. It also displayed an HER overpotential of 289.7 mV at −10 mA cm^−2^, with a Tafel slope of 247.8 mV dec^−1^. Furthermore, the material demonstrated excellent stability with a low water-splitting voltage of 1.96 V at 50 mA cm^−2^ after 14 h of cycling. We also evaluated the catalyst’s performance in seawater, where it exhibited an OER overpotential of 389.5 mV and a Tafel slope of 236.4 mV dec^−1^ at 30 mA cm^−2^. For HER, the overpotential and Tafel slope were 203.5 mV and 113.6 mV dec^−1^ at −10 mA cm^−2^, respectively, representing significant decreases of 86.2 mV and 134.2 mV dec^−1^ compared with alkaline conditions. During the process, ion migration and adsorption likely play key roles, with the larger interlayer spacing of Mn-Co_1.29_Ni_1.71_O_4_ facilitating the diffusion of OH^−^ and Cl^−^ ions to the active centers.

## 2. Results and Discussion

**Figure 1:**
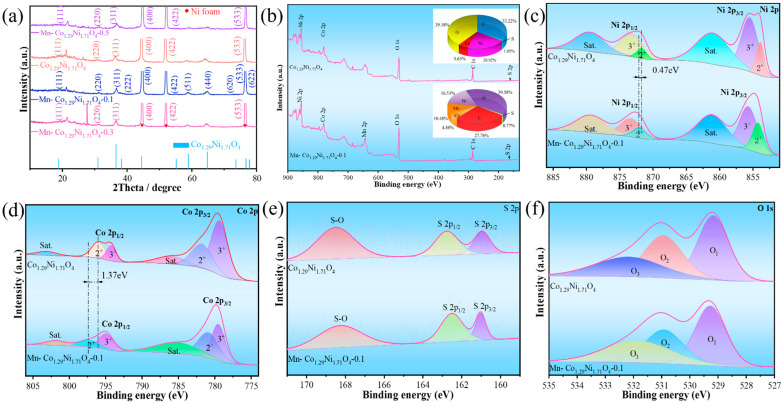
Structural characterization of the prepared catalyst: (**a**) XRD spectrum, (**b**) full XRD spectrum of the sample, (**c**) Ni 2p, (**d**) Co 2p, (**e**) S 2p, (**f**) O 1s.

The crystalline phase structure and composition of the samples were determined using X-ray diffraction (XRD). Figure 1a shows the XRD characterization results of Co_1.29_Ni_1.71_O_4_ materials doped with different concentrations of Mn ions. The orange, blue, pink, and purple lines represent Mn-Co_1.29_Ni_1.71_O_4_ doped with 0 mM Mn ions, 0.1 mM Mn ions, 0.3 mM Mn ions, and 0.5 mM Mn ions, respectively. The red diamond markers in the spectrum indicate the characteristic peaks of foam nickel. The main peaks at 18.8°, 31.0°, 36.6°, 38.3°, 44.5°, 55.1°, 58.9°, 64.8°, 73.4°, 76.7°, and 77.8° correspond to the (111), (220), (311), (222), (400), (422), (511), (440), (620), (533), and (622) crystal planes of Co_1.29_Ni_1.71_O_4_, respectively. These peaks indicate that the Co_1.29_Ni_1.71_O_4_ crystal has a complex cubic structure, confirming the successful synthesis of Mn-Co_1.29_Ni_1.71_O_4_ materials in this experiment. Additionally, the distribution of these peaks reveals that all the materials exhibit excellent crystallinity, meaning that the Mn-Co_1.29_Ni_1.71_O_4_ materials maintain their original crystal structure without significant structural distortion during the preparation process.

The chemical element composition and valence state of the prepared samples were analyzed using X-ray photoelectron spectroscopy (XPS). The binding energy of the XPS spectra was calibrated using the C 1s peak at 284.8 eV, and the results are shown in Figure 1b. It can be observed that the Co_1.29_Ni_1.71_O_4_ sample contains Ni, Co, O, C, and S elements, while the Mn-Co_1.29_Ni_1.71_O_4_ sample contains Mn and the above-mentioned elements, confirming the successful incorporation of Mn into Co_1.29_Ni_1.71_O_4_ [19,20]. Additionally, the element pie chart indicates that the Mn content is 10.48% [21]. The XPS spectra of Ni 2p in Figure 1c show three peaks. The peaks at 855.8 eV and 854.2 eV are attributed to Ni^3+^ and Ni^2+^, respectively, while the peak at 861.3 eV is defined as a satellite peak [22,23]. Furthermore, compared with the Co_1.29_Ni_1.71_O_4_ sample, the Mn-Co_1.29_Ni_1.71_O_4_-0.1 catalyst exhibits a shift to higher binding energies, indicating a strong electronic interaction between Ni and Co atoms, which is beneficial for enhancing the adsorption of OH^−^ [24].

Figure 1d shows the Co 2p spectra of Co_1.29_Ni_1.71_O_4_ and Mn-Co_1.29_Ni_1.71_O_4_-0.1 samples. The peaks at 779.7 eV and 795.1 eV correspond to Co 2p_3/2_ and Co 2p_1/2_, both representing spin states of Co [24]. In the Co 2p_3/2_ region, the binding energy near 779.6 eV corresponds to Co^3+^, while the binding energy near 781 eV is attributed to Co^2+^ [25]. The peaks near 785.5 eV and 801.7 eV correspond to satellite peaks. Comparing the Co 2p spectra before and after Mn doping reveals a shift to higher binding energies by approximately 1.37 eV, indicating the presence of both Co^3+^ and Co^2+^ [26]. The O 1s spectrum, as shown in Figure 1e, can be fitted to three characteristic peaks. The peak at 529.2 eV corresponds to metal–oxygen bonds, the peak at 530.9 eV is attributed to surface low-coordination oxygen ions, and the peak at 531.9 eV is related to adsorbed water [27]. The S 2p spectrum, shown in Figure 1f, reveals peaks at 160.9 eV and 162.5 eV for S 2p_3/2_ and S 2p_1/2_, respectively. The peak at 168.2 eV corresponds to the oxidized state, indicating strong electronic effects of the catalyst [28].

**Figure 2:**
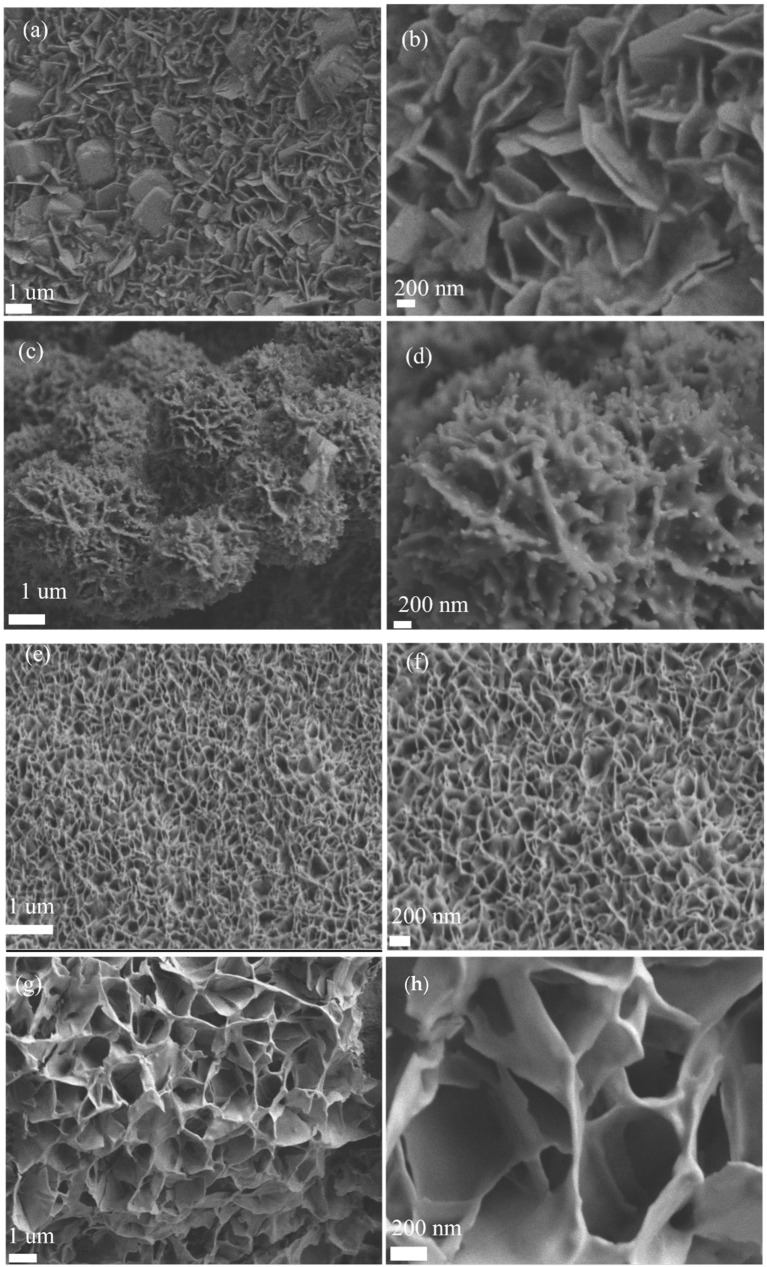
SEM morphology of the samples: (**a**,**b**) SEM morphology of the Co_1.29_Ni_1.71_O_4_ sample, (**c**,**d**) SEM morphology of the Mn-Co_1.29_Ni_1.71_O_4_-0.1 sample, (**e**,**f**) SEM morphology of the Mn-Co_1.29_Ni_1.71_O_4_-0.3 sample, (**g**,**h**) SEM morphology of the Mn-Co_1.29_Ni_1.71_O_4_-0.5 sample.

Scanning electron microscopy (SEM) images (Figure 2a–h) show the nanoflake morphology of Mn-Co_1.29_Ni_1.71_O_4_ with nanoparticles. The Mn doping ratio was controlled by varying the amount of manganese acetate added during the preparation. SEM images of Mn-Co_1.29_Ni_1.71_O_4_ at different doping levels show the shape of the nanoflakes at higher magnification (200 nm), while the thickness of the nanoflakes can be observed at lower magnification (1 µm). With the increase in Mn doping, the gap between the nanoflakes decreases, which leads to a reduction in electronic conductivity. When the Mn doping level reaches 0.5 mM, the annealing process causes the collapse of most of the flaky structures. However, the optimal doping level is 0.1 mM, as it ensures that the nanoflakes do not collapse and also form a certain amount of nanoparticles, increasing the catalyst’s surface area and facilitating fast electron transfer.

**Figure 3:**
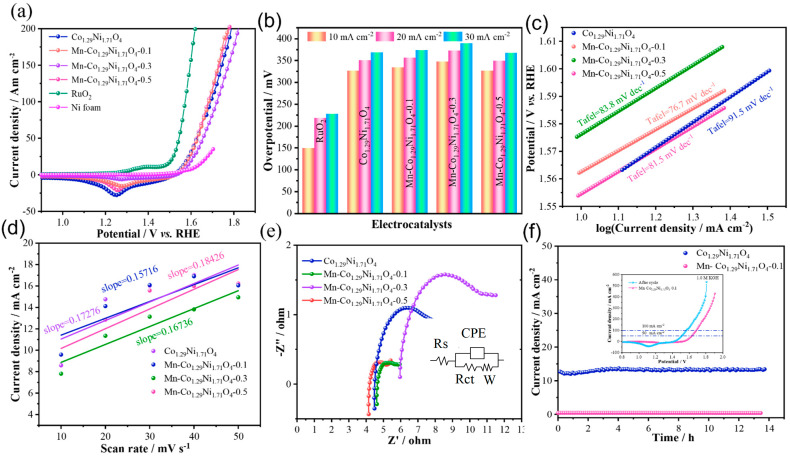
OER performance of the prepared samples: (**a**) LSV curve of the catalyst, (**b**) Overpotential bar chart of the catalyst, (**c**) Tafel slope of the catalyst, (**d**) Cdl value of the catalyst, (**e**) Impedance spectrum of the catalyst, (**f**) Stability of the catalyst.

To evaluate the electrocatalytic performance of the prepared samples, the OER performance of the materials in 1.0 M KOH electrolyte was first assessed through Linear Sweep Voltammetry (LSV). Figure 3a shows the LSV curves of the samples, where an oxidation peak can be observed around 1.25 V [29]. Among the four catalysts with different Mn doping levels, Mn-Co_1.29_Ni_1.71_O_4_-0.1 exhibits the best OER activity, with an overpotential of 373.3 mV at a current density of 30 mA cm^−2^. The Co_1.29_Ni_1.71_O_4_ sample has an overpotential of 368.3 mV at 30 mA cm^−2^, while Mn-Co_1.29_Ni_1.71_O_4_-0.3 and Mn-Co_1.29_Ni_1.71_O_4_-0.5 have overpotentials of 389.3 mV and 367.3 mV at 30 mA cm^−2^, respectively, as shown in Figure 3b. To compare the catalytic activity of the samples, the performance of the precious metal catalyst RuO_2_ was also measured, with an overpotential of 227.3 mV at 30 mA cm^-2^. To further demonstrate the advantages of Mn doping in Co_1.29_Ni_1.71_O_4_ electrocatalysts, the corresponding Tafel plots were analyzed to examine the OER kinetics. As shown in Figure 3c, the Tafel slope of Mn-Co_1.29_Ni_1.71_O_4_-0.1 is 76.7 mV dec^−1^, which is lower than that of Co_1.29_Ni_1.71_O_4_ (91.5 mV dec^−1^), Mn-Co_1.29_Ni_1.71_O_4_-0.3 (83.8 mV dec^−1^), and Mn-Co_1.29_Ni_1.71_O_4_-0.5 (81.5 mV dec^−1^), indicating that appropriate Mn doping can enhance OER kinetics [30]. Linear fitting of these curves yielded the slope, which corresponds to the C_dl. The C_dl of the catalyst is proportional to its electrochemical surface area (ECSA), which generally correlates with the material performance.

The electrochemical surface area obtained from the double-layer capacitance is shown in Figure 3d. The Cdl of Mn-Co_1.29_Ni_1.71_O_4_-0.1 is 0.157 mF cm^−2^, which is lower than that of Co_1.29_Ni_1.71_O_4_ (0.173 mF cm^−2^), Mn-Co_1.29_Ni_1.71_O_4_-0.3 (0.167 mF cm^−2^), and Mn-Co_1.29_Ni_1.71_O_4_-0.5 (0.184 mF cm^−2^). It is noteworthy that while the ECSA of Mn-Co_1.29_Ni_1.71_O_4_-0.1 is lower than the other samples, its overpotential and Tafel slope are also lower, suggesting that Mn-Co_1.29_Ni_1.71_O_4_-0.1 exhibits higher intrinsic activity.

To further investigate the influence of Mn doping on the electron transfer during OER, electrochemical impedance spectroscopy (EIS) tests were conducted of the as-prepared Co_1.29_Ni_1.71_O_4_, Mn-Co_1.29_Ni_1.71_O_4_-0.1, Mn-Co_1.29_Ni_1.71_O_4_-0.3, and Mn-Co_1.29_Ni_1.71_O_4_-0.5 samples, as shown in Figure 3e. The results indicate that the transport resistance of Mn-Co_1.29_Ni_1.71_O_4_-0.1 is lower than that of Mn-Co_1.29_Ni_1.71_O_4_-0.5. To evaluate the long-term stability of Mn-Co_1.29_Ni_1.71_O_4_-0.1 for OER in an alkaline environment, chronoamperometric measurements were conducted at a constant current density of 10 mA cm^−2^ in 1.0 M KOH, as shown in Figure 3f. After 14 h of continuous testing, Mn-Co_1.29_Ni_1.71_O_4_-0.1 showed superior long-term durability compared with Co_1.29_Ni_1.71_O_4_ under the same conditions, demonstrating that Mn doping can enhance the material’s conductivity and stability [30].

**Figure 4:**
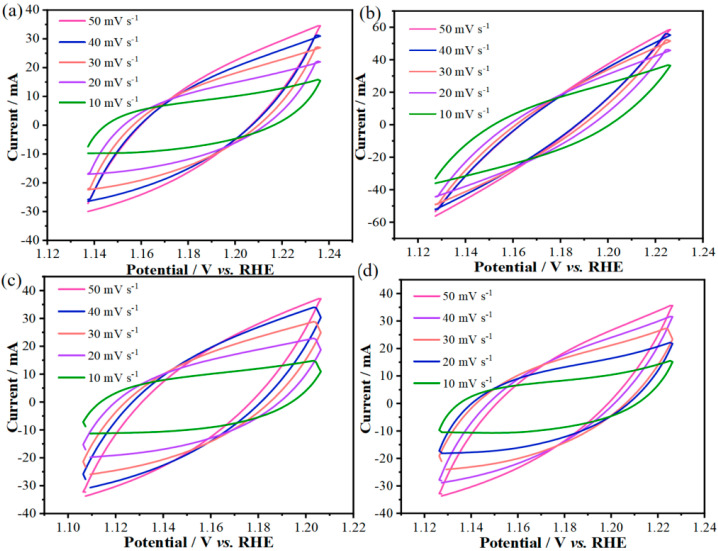
CV curves of the prepared samples for OER: (**a**) CV curve of the Co_1.29_Ni_1.71_O_4_ sample, (**b**) CV curve of the Mn-Co_1.29_Ni_1.71_O_4_-0.1 sample, (**c**) CV curve of the Mn-Co_1.29_Ni_1.71_O_4_-0.3 sample, (**d**) CV curve of the Mn-Co_1.29_Ni_1.71_O_4_-0.5 sample.

To assess the catalyst activity, the double-layer capacitance (C_dl) of different electrodes was calculated. Cyclic voltammetry (CV) tests were performed on the as-prepared Co_1.29_Ni_1.71_O_4_, Mn-Co_1.29_Ni_1.71_O_4_-0.1, Mn-Co_1.29_Ni_1.71_O_4_-0.3, and Mn-Co_1.29_Ni_1.71_O_4_-0.5 samples at different scan rates (from 10 mV s^−1^ to 50 mV s^−1^), as shown in Figure 4a–d. It can be seen that the CV curve areas of all the samples increase with the scan rate. 

**Figure 5:**
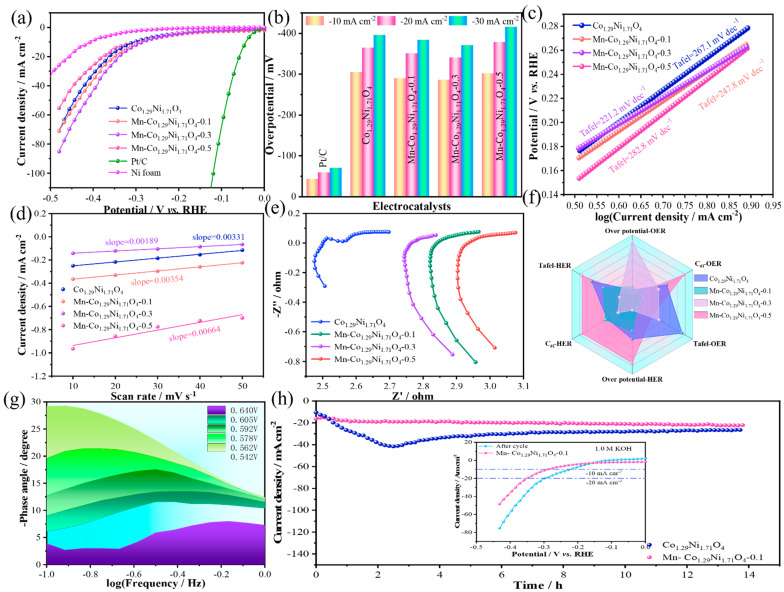
HER performance of the samples: (**a**) LSV curve of the catalyst, (**b**) Overpotential bar chart of the catalyst, (**c**) Tafel slope of the catalyst, (**d**) Cdl value of the catalyst, (**e**) AC of the catalyst, (**f**) Radar chart of the catalyst, (**g**) Nyquist plot of the catalyst, (**h**) Stability of the catalyst.

The HER performance evaluation of the prepared catalysts was conducted under the same conditions. Figure 5a shows the linear sweep voltammetry (LSV) curves of the samples and noble metals, with a scan rate of 5 mV/s. The results indicate that the overpotential of the noble metal Pt/C at a current density of −10 mA/cm^2^ is 43.4 mV, which is lower than that of Co_1.29_Ni_1.71_O_4_ (304.7 mV @ −10 mA/cm^2^), Mn-Co_1.29_Ni_1.71_O_4_-0.1 (289.7 mV @ −10 mA/cm^2^), Mn-Co_1.29_Ni_1.71_O_4_-0.3 (285.7 mV @ −10 mA/cm^2^), and Mn-Co_1.29_Ni_1.71_O_4_-0.5 (301.7 mV @ −10 mA/cm^2^) catalysts in Figure 5b. To further investigate the HER reaction kinetics, the corresponding Tafel slopes were calculated from the polarization curves. A smaller Tafel slope indicates a faster HER kinetic process as the reaction progresses and an improvement in the reaction rate limitation at the catalyst surface [31]. As shown in Figure 5c, the Tafel slope of Co_1.29_Ni_1.71_O_4_ is 267.1 mV/dec, which is higher than that of Mn-Co_1.29_Ni_1.71_O_4_-0.1 (247.8 mV/dec), Mn-Co_1.29_Ni_1.71_O_4_-0.3 (221.2 mV/dec), and Mn-Co_1.29_Ni_1.71_O_4_-0.5 (282.8 mV/dec). These data clearly demonstrate that the electron transfer rate at the catalyst surface increases initially and then decreases as the Mn doping content increases. On the other hand, electrochemical surface area (ECSA) is also an important parameter for evaluating catalyst performance, reflecting the effective area involved in the catalytic reaction and its intrinsic activity. Since ECSA is proportional to the double-layer capacitance (C_dl_), the Cdl of the reaction samples was calculated to assess the ECSA of the catalysts. The linear fitting of the current densities at different scan rates gives the slope of the fitting curve, which corresponds to the double-layer capacitance (C_dl_) of the sample [32]. The electrochemical surface area obtained from the electrochemical double-layer capacitance is shown in Figure 5d. The C_dl_ of Co_1.29_Ni_1.71_O_4_ is 0.00331 mF/cm^2^, Mn-Co_1.29_Ni1.71O_4_-0.1 (0.00354 mF/cm^2^), Mn-Co_1.29_Ni_1.71_O_4_-0.3 (0.00189 mF/cm^2^), and Mn-Co_1.29_Ni_1.71_O_4_-0.5 (0.0664 mF/cm^2^). As the Mn doping content increases, a trend in the change in Cdl values for different materials can be observed, which is almost consistent with the HER activity sequence. Among them, Mn-Co_1.29_Ni_1.71_O_4_-0.5 has the highest C_dl_ value, indicating the largest active surface area.

Figure 5f is a radar plot based on the overpotentials (OER and HER), electroactivity (Cdl), and Tafel slopes of the materials. From the radar plot, it can be seen that Mn-Co_1.29_Ni_1.71_O_4_-0.1 exhibits the best overall performance for water splitting. The Bode plot (Figure 5g) shows that the charge transfer at the catalyst/electrolyte interface occurs in the low-frequency range of 10^−1^ to 10^0^, with the peak values decreasing more rapidly as the potential increases, revealing a significant enhancement of the OER activity of Mn-Co_1.29_Ni_1.71_O_4_-0.1. To evaluate the long-term stability of Mn-Co_1.29_Ni_1.71_O_4_-0.1 for HER in an alkaline environment, a chronoamperometric measurement was performed at a constant current density of 10 mA/cm^2^ in 1.0 M KOH, as shown in Figure 5h. After 14 h of continuous testing, Mn-Co_1.29_Ni_1.71_O_4_-0.1 showed superior long-term durability compared with Co_1.29_Ni_1.71_O_4_ under the same conditions, suggesting that Mn doping can improve the material’s conductivity and stability.

**Figure 6:**
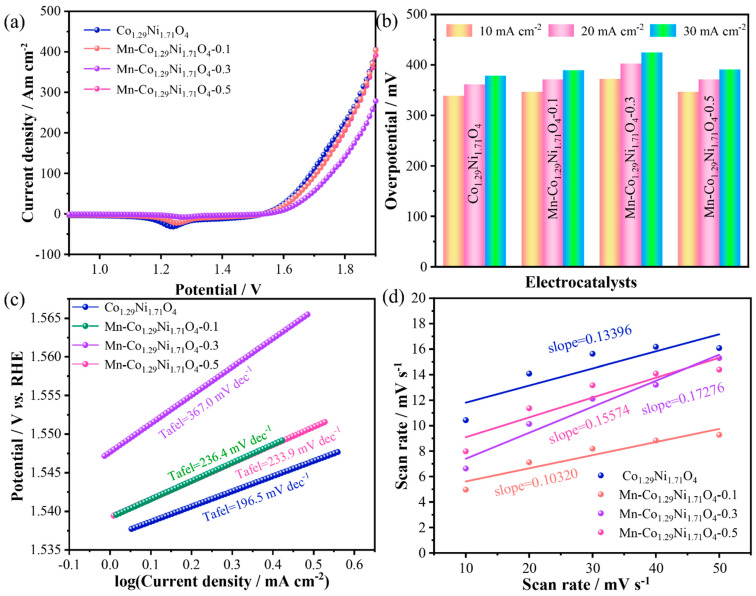
Seawater OER performance of the prepared samples: (**a**) LSV curve of the catalyst, (**b**) Bar chart of the catalyst’s overpotential, (**c**) Tafel curve of the catalyst, (**d**) Cdl value of the catalyst.

To evaluate the electrocatalytic performance of the prepared samples in seawater, the OER performance of the materials was first assessed through LSV. Figure 6a shows the LSV curves of the samples, where an oxidation peak is observed around 1.24 V. Among the four catalysts with different Mn doping levels, Mn-Co_1.29_Ni_1.71_O_4_-0.1 exhibited the best OER activity with an overpotential of 389.5 mV at a current density of 30 mA cm^−2^, superior to the other Mn-doped catalysts Mn-Co_1.29_Ni_1.71_O_4-_0.3 (424.5 mV@30 mA cm^−2^) and Mn-Co_1.29_Ni_1.71_O_4_-0.5 (390.5 mV@30 mA cm^−2^),as shown in Figure 6b. To compare the catalytic activity of the samples and further demonstrate the advantage of Mn doping in Co_1.29_Ni_1.71_O_4_ electrocatalysts, the corresponding Tafel plots were calculated from the LSV data to analyze the OER kinetics. As shown in Figure 6c, the Tafel slope of Mn-Co_1.29_Ni_1.71_O_4_-0.1 is 236.4 mV dec^−1^, while Mn-Co_1.29_Ni_1.71_O_4_-0.3 is 367.0 mV dec^−1^, and Mn-Co_1.29_Ni_1.71_O_4_-0.5 is 233.9 mV dec^−1^. This indicates that moderate Mn doping can promote OER kinetics. To evaluate the catalyst activity, the electrochemical double-layer capacitance (Cdl) of different electrodes was calculated. The C_dl_ of the catalyst is proportional to its electrochemical surface area (ECSA), which generally correlates with the material performance.The electrochemical surface area calculated from the electrochemical double-layer capacitance is shown in Figure 6d. The C_dl_ of Mn-Co_1.29_Ni_1.71_O_4_-0.1 is 0.103 mF cm^−2^, lower than that of Co_1.29_Ni_1.71_O_4_ (0.134 mF cm^−2^), Mn-Co_1.29_Ni_1.71_O_4_-0.3 (0.173 mF cm^−2^), and Mn-Co_1.29_Ni_1.71_O_4_-0.5 (0.156 mF cm^−2^). It is noteworthy that while the ECSA of Mn-Co_1.29_Ni_1.71_O_4_-0.1 is lower than the other samples, its overpotential and Tafel slope are also lower, indicating that Mn-Co_1.29_Ni_1.71_O_4_-0.1 exhibits higher intrinsic activity.

**Figure 7:**
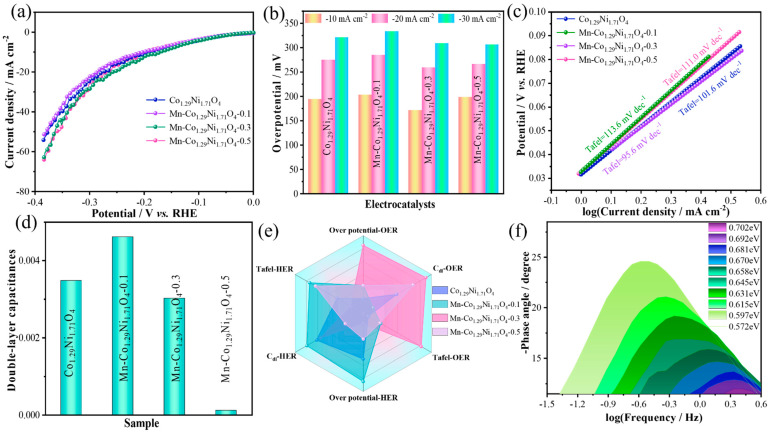
HER performance of the prepared samples in seawater: (**a**) LSV curve of the catalyst, (**b**) Bar chart of the catalyst overpotential, (**c**) Tafel slope of the catalyst, (**d**) Cdl value of the catalyst, (**e**) Radar chart of the catalyst, (**f**) Nyquist plot of the catalyst.

The HER performance of the prepared catalysts was evaluated under the same conditions. Figure 7a shows the LSV curves of the samples. The results with a scan rate of 5 mV s^−1^ indicate that at a current density of −10 mA cm^−2^, the overpotential of Co_1.29_Ni_1.71_O_4_ is 194.5 mV, Mn-Co_1.29_Ni_1.71_O_4_-0.1 is 203.5 mV, Mn-Co_1.29_Ni_1.71_O_4_-0.3 is 171.8 mV, and Mn-Co_1.29_Ni_1.71_O_4_-0.5 is 198.5 mV(Figure 7b). To further investigate the HER reaction kinetics, the corresponding Tafel slopes were calculated from the polarization curves in Figure 7c. A smaller Tafel slope indicates faster HER kinetics as the reaction proceeds, improving the surface reaction rate. The Tafel slope of Mn-Co_1.29_Ni_1.71_O_4_-0.3 is 95.6 mV dec^−1^, lower than that of Co_1.29_Ni_1.71_O_4_ (101.6 mV dec^−1^), Mn-Co_1.29_Ni_1.71_O_4_-0.1 (113.6 mV dec^−1^), and Mn-Co_1.29_Ni_1.71_O_4_-0.5 (111.0 mV dec^−1^). These data demonstrate that in seawater, the electron transfer rate on the catalyst surface decreases with increasing Mn doping content at first and then increases, showing a trend of first decreasing and then increasing. On the other hand, the electrochemical surface area (ECSA) is an important parameter for evaluating catalyst performance, reflecting the effective area and intrinsic activity involved in the catalytic reaction. Since ECSA is proportional to double-layer capacitance (Cdl), the ECSA of the catalysts was evaluated by calculating the Cdl of the samples. The slope of the fitting curve is the value of the double-layer capacitance (Cdl).

The electrochemical surface area obtained from the electrochemical double-layer capacitance is shown in Figure 7d. The Cdl of Co_1.29_Ni_1.71_O_4_ is 0.00331 mF cm^−2^, Mn-Co_1.29_Ni_1.71_O_4_-0.1 is 0.00354 mF cm^−2^, Mn-Co_1.29_Ni_1.71_O_4_-0.3 is 0.00189 mF cm^−2^, and Mn-Co_1.29_Ni_1.71_O_4_-0.5 is 0.000116 mF cm^−2^. With the increase in Mn doping, the trend in the change in the Cdl values for the different materials can be observed. Among them, Mn-Co_1.29_Ni_1.71_O_4_-0.1 has the highest Cdl value, corresponding to the largest active surface area. Figure 7e is a radar chart that plots the overpotential, electroactivity (Cdl), and Tafel slope of the materials for OER and HER. According to the radar chart, the overpotentials, Tafel slopes, and OER and HER overpotentials for Co_1.29_Ni_1.71_O_4_, Mn-Co_1.29_Ni_1.71_O_4_, Mn-Co_1.29_Ni_1.71_O_4_-0.3, and Mn-Co_1.29_Ni_1.71_O_4_-0.5 are shown. A comprehensive comparison of these indicators reveals that Mn-Co_1.29_Ni_1.71_O_4_-0.1 exhibits the highest overall performance, with the best water electrolysis performance. The Nyquist plot (Figure 7f) indicates that charge transfer at the catalyst/electrolyte interface occurs in the low-frequency range of 10–1.5~100.6 Hz. As the potential increases, these peaks decrease more rapidly, revealing that the OER activity of Mn-Co_1.29_Ni_1.71_O_4_-0.1 is significantly enhanced [33,34].

**Figure 8:**
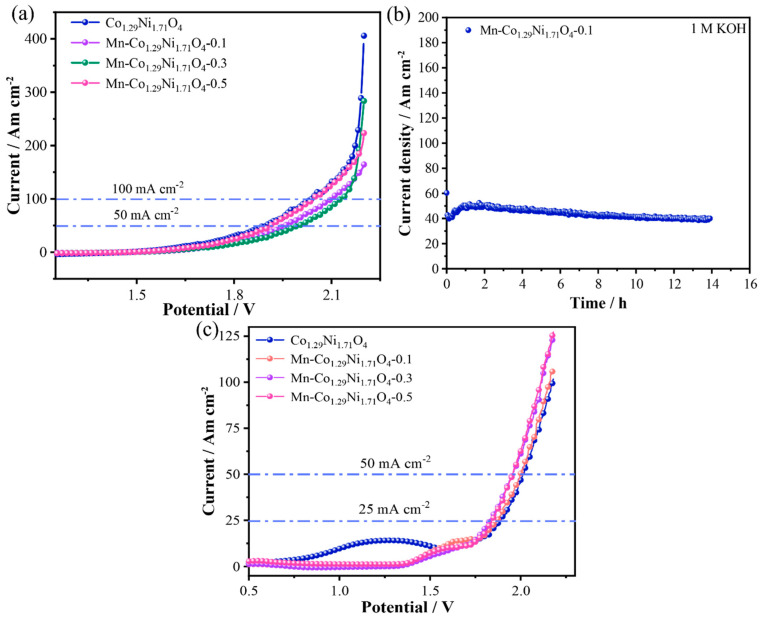
Overall water splitting performance of the prepared samples: (**a**) LSV of overall water splitting, (**b**) Catalyst stability, (**c**) LSV of overall water splitting in seawater.

Based on the above electrochemical test results, the Mn-Co_1.29_Ni_1.71_O_4_-0.1 sample shows excellent OER and HER performance in an alkaline medium (1.0 M KOH). Figure 8a shows the LSV curves. At current densities of 50 mA cm^−2^ and 100 mA cm^−2^, the overall water splitting potential of the Mn-Co_1.29_Ni_1.71_O_4_-0.1 electrode material is 1.96 V and 2.10 V, respectively, which is lower than that of the Mn-Co_1.29_Ni_1.71_O_4_-0.3 catalyst (2.00 V and 2.12 V). During the electrolysis process, gas bubbles are visibly generated on both sides of the electrodes. The durability of the catalyst is an important indicator for assessing its performance. As shown in Figure 8b, the stability test demonstrates that after 14 h of cycling, the Mn-Co_1.29_Ni_1.71_O_4_-0.1 catalyst exhibits excellent stability, with no significant decrease in current density. Additionally, the Mn-Co_1.29_Ni_1.71_O_4_-0.1 sample shows good OER and HER performance in seawater. Figure 8c shows the LSV curves. At current densities of 50 mA cm^−2^ and 100 mA cm^−2^, the seawater overall water splitting potential of the Mn-Co_1.29_Ni_1.71_O_4_-0.1 electrode material is 1.87 V and 2.00 V, respectively, which is lower than that of the Co_1.29_Ni_1.71_O_4_ catalyst (1.89 V and 2.02 V). During the electrolysis process, gas bubbles are clearly visible on both sides of the electrodes.

## 3. Experimental Section

All reagents are analytically pure and do not require further purification. First, a 5 × 7 cm^2^ nickel foam is cut and immersed in anhydrous ethanol, followed by ultrasonic cleaning for 30 min. The foam is then alternately sonicated in deionized water and ethanol, repeating the process three times to thoroughly remove surface oxides and impurities. After cleaning, the foam is placed in a vacuum drying oven at 60 °C and dried for at least 24 h. Then, a solution is prepared by dissolving 1 mM nickel nitrate, 2 mM cobalt nitrate, 5 mM urea, and 6 mM ammonium fluoride in 60 mL of deionized water. The pretreated nickel foam and this solution are transferred into a reaction vessel and maintained at 120 °C for 6 h. After cooling to room temperature, the precursor is removed, thoroughly washed with deionized water, and annealed at 350 °C for 2 h, yielding the Co_1.29_Ni_1.71_O_4_ samples.

### 3.1. Synthesis of Mn-Co_1.29_Ni_1.71_O_4_ Samples

Similar to the Co_1.29_Ni_1.71_O_4_ synthesis, 4 × 5 cm nickel foam is sonicated in ethanol and deionized water for 30 min, then dried overnight at 60 °C. A solution is prepared by dissolving 1 mM nickel nitrate, 2 mM cobalt nitrate, 5 mM urea, 6 mM ammonium fluoride, and varying amounts of manganese acetate (0, 0.1, 0.3, and 0.5 mM) in 60 mL deionized water. The pretreated nickel foam and the solution are transferred into a reaction vessel and maintained at 120 °C for 6 h. After cooling to room temperature, the precursor is washed thoroughly with deionized water and annealed at 350 °C for 2 h to obtain Mn-Co_1.29_Ni_1.71_O_4_ samples. Based on the amount of manganese acetate added, the as-prepared samples are named Mn-Co_1.29_Ni_1.71_O_4_-0.1, Mn-Co_1.29_Ni_1.71_O_4_-0.3, and Mn-Co_1.29_Ni_1.71_O_4_-0.5, respectively.

### 3.2. Electrochemical Performance Measurements

The electrocatalytic performance of all samples was measured using an electrochemical workstation (CHI660e). The OER and HER tests were conducted in a three-electrode system. The prepared catalysts served as the working electrodes, with Hg/HgO as the reference electrode. Graphite rods and platinum plates were used as the counter electrodes for HER and OER, respectively. A 1.0 M KOH solution (pH = 13.7) was used as the electrolyte. Overall water splitting performance was tested in a two-electrode system at a scan rate of 5 mV s^−1^, which includes cyclic voltammetry, chronopotentiometry, and linear sweep voltammetry (LSV) curves with 90% IR compensation. Additionally, electrochemical impedance spectroscopy (EIS) was carried out with an AC voltage amplitude of 5 mV and a frequency range of 0.01 Hz to 100 kHz. Potentials were converted to a reversible hydrogen electrode (RHE) using the Nernst equation: E_RHE_ = E_Hg/HgO_ + 0.059 × pH + 0.098. The OER overpotential (η) was calculated as η = E_RHE_ − 1.23 V.

## 4. Conclusions

In summary, Mn-Co_1.29_Ni_1.71_O_4_ electrocatalysts were synthesized using a hydrothermal method. Due to their unique nanostructure, the internal nanosheet layers interweave, providing a large specific surface area and thus offering numerous electrochemical active sites for the electrocatalytic reaction. The catalyst exhibits excellent OER and HER performance in both alkaline water and seawater electrolytes. In 1.0 M KOH electrolyte, the prepared Mn-Co_1.29_Ni_1.71_O_4_ catalyst demonstrates good OER performance with an oxygen evolution overpotential of 334.3 mV at a current density of 10 mA cm^−2^ and a corresponding overpotential of 373.3 mV at 30 mA cm^−2^. It also shows a Tafel slope of 76.7 mV dec^−1^ and good durability. As a hydrogen evolution electrocatalyst, it exhibits a hydrogen evolution overpotential of 203.5 mV at −10 mA cm^−2^ and a Tafel slope of 113.6 mV dec^−1^. When used in water splitting, the corresponding water splitting voltage is 1.96 V at 50 mA cm^−2^. In seawater, the catalyst also demonstrates superior OER performance, with an oxygen evolution overpotential of 389.5 mV at a current density of 30 mA cm^−2^ and a Tafel slope of 236.4 mV dec^−1^. As a hydrogen evolution electrocatalyst, it shows a hydrogen evolution overpotential of 203.5 mV at −10 mA cm^−2^ and a Tafel slope of 113.6 mV dec^−1^. In seawater, when used for water splitting, the corresponding water splitting voltage is 1.87 V at 50 mA cm^−2^. This study provides new insights into the rational design and preparation of efficient electrocatalysts, laying the foundation for the development of more efficient alkaline water/seawater electrocatalytic materials.

## Data Availability

Data is contained within the article.
